# Bayesian modeling of *Escherichia coli* contamination in household drinking water in Bangladesh: evidence from the Multiple Indicator Cluster Survey 2019

**DOI:** 10.1093/inthealth/ihaf138

**Published:** 2025-12-05

**Authors:** Iqramul Haq, Azizur Rahman, Mst Romana Akter, Delower Hossain, Diego Nobrega

**Affiliations:** Department of Agricultural Statistics, Sher-e-Bangla Agricultural University, Dhaka-1207, Bangladesh; Faculty of Veterinary Medicine, University of Calgary, Calgary, AB T2N 1N4, Canada; Department of Statistics and Data Science, Jahangirnagar University, Savar, Dhaka-1342, Bangladesh; Department of Community Health Sciences, University of Manitoba, Winnipeg, MB R3A 0W5, Canada; Department of Statistics, Mawlana Bhashani Science and Technology University, Santosh, Tangail – 1902, Bangladesh; Department of Medicine and Public Health, Sher-e-Bangla Agricultural University, Dhaka- 1207, Bangladesh; Department of Veterinary Medicine and Animal Sciences (DIVAS), Università degli Studi di Milano, Lodi 26900, Italy; Faculty of Veterinary Medicine, University of Calgary, Calgary, AB T2N 1N4, Canada

**Keywords:** censored, *E. coli*, fecal contamination, sanitation, sociodemographic, water

## Abstract

**Background:**

From a public health standpoint, there is merit in determining the levels of *Escherichia coli* in drinking water, but surveillance datasets often report censored values that may hinder traditional statistical analysis. This study aims to identify sociodemographic factors associated with the presence of *E. coli* in household drinking water in Bangladesh using Bayesian models for censored data, utilizing data from 6069 households in the Multiple Indicator Cluster Survey 2019.

**Methods:**

In terms of censoring, we considered two different Bayesian regression strategies: Bayesian Tobit Poisson regression and Bayesian Censored Generalized Poisson regression.

**Results:**

The Bayesian Censored Generalized Poisson regression model was identified as the optimal model for analyzing household fecal contamination. Regression analysis revealed significant associations between household *E. coli* levels and various factors including division, livestock ownership, location of water sources, treatment of drinking water, household head education, wealth index, source of drinking water, place of handwashing and toilet facility. Households using tube wells had lower *E. coli* levels than those using other sources. Furthermore, households using pit latrines had 1.03 times higher contamination levels than those using flush latrines.

**Conclusions:**

Levels of fecal contamination in household water in Bangladesh were alarming. Our findings underscore the need for targeted policy interventions in specific population segments to address household fecal contamination, highlighting the link between sociodemographic and environmental factors with *E. coli* levels in drinking water.

## Introduction

*Escherichia coli* is a ubiquitous bacteria present almost everywhere in our environment, including water, soil, foods and the intestines of living mammals. *E. coli* is very diverse, and most strains are harmless to humans. However, specific strains of *E. coli* are pathogenic to humans, and may cause important clinical syndromes such as gastrointestinal, lower-respiratory tract, bloodstream and urinary infections.^1^ Because *E. coli* colonization with certain strains can be associated with an increased risk of clinical infections, water and food products should be free of *E. coli* contamination.^2^

In addition to humans, *E. coli* also causes diseases in animals, including food-producing animals and wildlife.^3^  *E. coli* from animals may also carry antimicrobial resistance genes, with prevalence increasing due to the abuse and misuse of antibiotics.^4^ In Bangladesh, many antibiotics are sold without prescriptions.^5^ The transmission of multidrug-resistant *E. coli* from animals to humans is notably high,^6^ with prior studies reporting up to 100% resistance in poultry isolates, likely due to prolonged antimicrobial use in farming.^7^  *E. coli* has shown high acquired resistance to ciprofloxacin, with rates of 98% in fecal and 89% in environmental isolates.^8^ From 2014 to 2016, Enterotoxigenic *E. coli* (ETEC) strains in Bangladesh exhibited high levels of multidrug resistance, particularly to nalidixic acid (80.7%), macrolides (49.1–89.7%), ampicillin (57.9–69%) and trimethoprim/sulfamethoxazole (55.2%).^9^

The WHO guidelines on drinking water underscore the critical importance of safe water for human health.^10^ Detection of *E. coli* in water serves as an indicator of potential dissemination of waterborne diseases.^11^ Importantly, contaminated drinking water is a potential source of diarrheagenic *E. coli* strains. Studies have detected diarrheagenic *E. coli* in drinking water samples, indicating that water can serve as a vehicle for these pathogens.^12^ During the rainy season, the risk factors for fecal contamination of boiled drinking water included scooping water with a cup, using a visibly dirty container and having a household size of ≥5 members.^13^

Additionally, the relationship between travel and *E. coli* colonization has been demonstrated.^14^ Interestingly, consuming meals with locals, receiving antibiotic treatment during travel and traveling for non-business purposes, were significantly linked to an increased risk of acquiring extended-spectrum beta-lactamase-producing *E. coli*. A study in Dhaka found that fecal contamination of water in urban areas was significantly higher in low-income neighborhoods compared with high-income areas. Furthermore, contamination levels were highest in open drains, surface water, floodwater, produce, soil and street food.^15^  *Escherichia coli* was detected in 19–64% of shallow tube wells, with higher levels during heavy rainfall.

There are many factors associated with *E. coli* contamination in household drinking water. For example, livestock ownership increases the risk of fecal contamination in drinking water.^16^ A positive correlation between *E. coli* contamination in drinking water and diarrheal episodes was demonstrated in young children in Bangladesh.^17–19^ Furthermore, associations between *E. coli* contamination and domestic hygiene practices in rural Bangladesh have also been demonstrated and suggest that covering stored food and maintaining water access can reduce fecal contamination.^20^ Additionally, water treatment practices are important elements to reduce the total coliform count in drinking water.^21,22^

The Multiple Indicator Cluster Survey (MICS) dataset from Bangladesh has been used to identify potential factors associated with *E. coli* contamination in household drinking water.^23,24^ Importantly, previous studies utilized models for multinomial or ordered data based on contamination thresholds. Conversely, there is merit in analyzing the actual *E. coli* counts in household drinking water. For one, dichotomizing continuous data reduces statistical power,^25^ which may be equivalent to discarding one-third of the data,^26^ as well as increasing the risk of false positives.^27^ It also masks variation within groups, misclassifies similar individuals as different and hides non-linear relationships, leading to potentially misleading conclusions.^25^ Continuous data are more flexible and represent variables that can take any value within a given range.^28^ While continuous data have many advantages, a common limitation is the introduction of censoring. Censoring occurs when only limited or incomplete quantitative data are available.^29^ When the dependent variable is censored, standard estimation methods may give inconsistent and biased results.^30^ For handling censored data, traditional statistical methods may introduce systematic bias.^31^ When the outcome variable is censored, Tobit-type regression models^32^ and censored regression models can be used.^33^ Tobit regression is particularly useful in such cases, as it incorporates censored observations to estimate parameters more accurately than traditional methods.^31^ However, although Tobit models handle censored data better than ordinary least squares (OLS) regression, they depend on strong distributional assumptions.^34^ For example, the Tobit model, like OLS, relies on strict assumptions of normality, proportionality and homoskedasticity, whose violations can produce similar biases to those seen in OLS.^34,35^ In this context, censored Poisson regression is another robust alternative, offering consistent estimates by accounting for censoring effects.^30^

The Bayesian approach can be used to treat censored data as additional parameters, offering advantages in the presence of censored data or inadequate sample size, and for nested/non-nested model comparisons.^36^ Bayesian modeling is robust in statistical inference, even with a large proportion of missing data.^37,38^

In this context, our goal is to employ Bayesian Tobit Poisson regression (BTPR) and Bayesian Censored Generalized Poisson regression (BCGPR) models to identify the potential sociodemographic risk factors associated with the presence of *E. coli* in drinking water in Bangladesh. Furthermore, we investigate potential sociodemographic characteristics associated with fecal contamination in Bangladeshi households. Our goal was to identify characteristics at the household level that are associated with levels of *E. coli* in drinking water that could potentially be used to improve facilities and mitigate the risk of diarrhea in household members.

## Methodology

### Data sources

We utilized data from the Bangladesh MICS. This survey was conducted by the Bangladesh Bureau of Statistics (BBS) for a global MICS program in 2019, with funding provided by UNICEF. The aim of the MICS program was to collect statistically significant and globally compatible data, which are essential in developing national, international and political policies, goals and commitments.

MICS utilized a two-staged cluster sampling method. The number of primary sampling units was 3220. A symmetric sample of 20 households was drawn from each part (rural and urban) of each of the 64 districts of Bangladesh. From these, a subsample of four households was randomly selected for water quality testing for arsenic in their drinking water. Out of these four, two households were further randomly chosen for *E. coli* testing. Respondents from each selected household were asked to provide ‘a glass of water which you would give a child to drink’ for the water quality test. These samples are referred to as point of use or household samples. The respondent provided water for *E. coli* testing, and it was collected in a Whirl-Pak bag. The water was flushed for 30 s before collecting samples from source sites, but not for hand-collected sources. The unsterilized water source may have contributed to some *E. coli* contamination in the tests, possibly due to unsanitary handling by tap users or tube well spouts users. They were given questionnaires related to water quality and a total of 12 251 houses were inspected for water quality testing. From this total, 6149 households were selected for *E. coli* testing using a list of eligible households, and 6069 (response rate of 99%) completed an interview and quality test for *E. coli*.^39^ All analyses were conducted using the weighted sample to ensure national representativeness.

**Figure 1.**
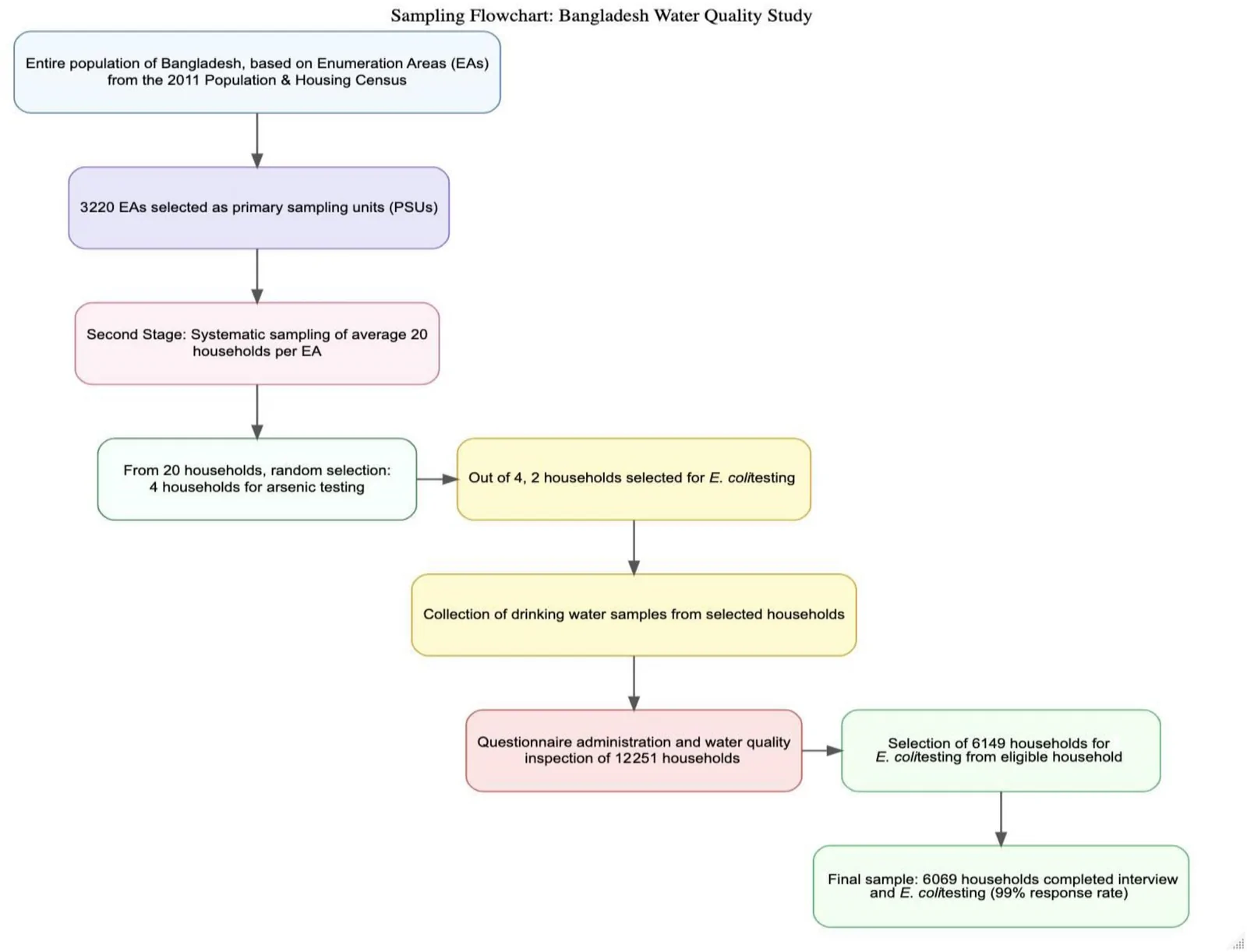
Flowchart of the sampling procedure for selection of households in the Bangladesh MICS 2019 water quality assessment. MICS: Multiple Indicator Cluster Survey.

The overall sampling procedure is illustrated in Figure 1.

### Independent variables

Variables were extracted from the interview and questionnaires are used in the analysis as explanatory variables, including the following: respondent’s administrative division (Barishal, Chattogram, Dhaka, Khulna, Mymensingh, Rajshahi, Rangpur, Sylhet), residential place (Urban, Rural), livestock ownership (Yes, No), location of water sources (Dwelling, Yard, Elsewhere), wealth index (Poor, Middle, Rich), education level of the head of the household (No education, Primary, secondary+), women education level (No education, Primary, secondary+), treatment of drinking water (Yes, No), mass media exposure (Yes, No), size of the household (<4, 4–5, ≥6), sources of drinking water (Piped water, Tube well, Other), Pit latrine or septic tank ever been emptied (Yes, No), places where handwashing is done (Dwelling, Yard/plot, Mobile object, No place), types of toilet facility (Flush, Pit latrine, Other) and sharing status of toilet facilities (Yes, No). In terms of sources of drinking water, we categorized other water sources (including dug wells, springs, carts, water kiosks, surface water such as rivers, dams, lakes, ponds, streams, canals and irrigation channels, as well as bottled water). The wealth index is a measure of a household’s wealth and uses 25 variables to capture long-term wealth through household assets, calculated using principal components analysis. The wealth index is derived from data on a household’s ownership of assets such as televisions and bicycles, housing construction materials and water access and sanitation facilities.^39^ Details of the independent variables and their classifications are provided in Supplemental Table 1.

### Dependent variables

The main outcome variable of interest was household fecal contamination level, represented by the number of *E. coli* colony-forming units (CFU) per 100 mL sample of drinking water. This is considered a reliable indicator of fecal contamination,^40^ and has been used before to assess drinking water quality.^41,42^ Our response variable is a count, consisting of non-negative integers, and is right-censored, with values >100 CFU/100 mL coded as 101.

### Enumeration of *E. coli*/*E. coli* testing

From each household, a 100 mL water sample was filtered using a 0.45 μm filter and inoculated onto media plates containing a chromogenic compound (X-gluc) to indicate growth of *E. coli*.^43^ After 24-h incubation at 37°C, the count of blue/green colonies was recorded. The water was then tested using membrane filtration with mTEC media. The enumeration methodology followed guidelines from the WHO listed under the Guidelines for Drinking Water Quality.^42^

### Statistical analysis

Frequency tables were used to summarize the results for each variable. The Poisson regression model provides a strong basis for count data analysis,^44,45^ with variants such as overdispersed, zero-inflated Poisson regression developed to handle additional data complexities.^46,47^ Prior to model fitting, we employed a Bayesian overdispersion test to assess count data dispersion. The test confirmed strong overdispersion, as evidenced by the observed dispersion ratio of 33.0, which greatly exceeded the posterior predictive mean dispersion of approximately 2.9 (Supplemental Figure 1). Additionally, the variance (1856.9) of the observed counts is much higher than the mean (43.6), thereby violating the Poisson equidispersion assumption. For overdispersed count data, the negative binomial (NB) model is a commonly used alternative, although it too may exhibit overdispersion under certain conditions.^48^ In the context of censored count data, estimation becomes more challenging due to the distortion of the mean–variance relationship; this relationship is typically unknown in censored datasets.^49,50^ Specifically, the observed mean and variance in censored data tend to be underestimated, making it difficult to determine whether the underlying distribution is overdispersed or underdispersed.^49^ As a result, the type of dispersion cannot be reliably inferred from observed values alone, and the impact of overdispersion on parameter estimates and SEs remains uncertain.^51^

To address these issues, researchers have explored a range of models. The Generalized Poisson Regression (GPR) model^52,53^ can accommodate both underdispersion and overdispersion, and Famoye and Wang extended it to censored data as Censored Generalized Poisson Regression.^49^ The GPR model extends the Poisson model by adding a dispersion parameter (ϕ), allowing it to handle both overdispersion (ϕ>0) and underdispersion (ϕ<0).^49,54^ Meanwhile, censored Poisson and NB models have been shown to outperform OLS and uncensored count models when censoring and overdispersion are present.^55^

For the right censoring, we attempted the following two different Bayesian regression models used for count data analysis: BTPR and BCGPR. Bayesian approaches have been proposed to address challenges such as under-reporting in count data. Bayesian frameworks can accommodate both a log-linear model for the Poisson rate and a logistic regression to relate reporting probabilities to covariates, using a combination of validation data and a larger fallible dataset.^56^

In both Bayesian models, posterior distribution estimation was performed using the Markov Chain Monte Carlo (MCMC) algorithm. The probabilistic programming language Stan (Stan Development Team, New York, NY, USA), implemented through R packages, was used to run MCMC.^57,58^ The *brms* (Paul-Christian Bürkner, University of Münster, Münster, Germany) and *rstan* (Stan Development Team, New York, NY, USA) packages were used to implement BTPR and BCGPR models in R, respectively.

Here, we used a prior distribution following a normal distribution with a mean of 0 and a variance of 10; that is, N(0,10) for the regression coefficients (class=‘b’). For BCGPR, we used a normal (0,1) prior on the dispersion parameter $\alpha ,\ {\rm which}\ {\mathrm{regularizes\ the\ overdispersion\ effect}}.{\mathrm{\ }} $This allows strong uncertainty but avoids extreme values. For MCMC convergence diagnostic we used rank-normalized $\hat{R} $ and considered chains to have converged if $\hat{R} < 1.01 $.^59^ We used four independent Markov chains to assess convergence. Each chain ran for 10 000 iterations, with the first 2000 iterations discarded as burn-in to remove initialization effects. This left 8000 effective iterations per chain, totaling 32 000 posterior samples across all chains.

#### Model selection

The leave-one-out cross-validation (LOO-CV) and widely or Watanabe applicable information criterion (WAIC) are techniques used to estimate the pointwise out-of-sample prediction accuracy from a Bayesian model and help assess model fit and generalizability.^60,61^ WAIC is a fully Bayesian method and preferable to deviance information criterion (DIC) in Bayesian modeling,^62^ especially with MCMC sampling, as it uses the entire posterior distribution and is asymptotically equivalent to Bayesian cross-validation, while DIC is not considered a singular model due to negative parameter estimates.^60^ Additionally, DIC is not fully Bayesian because it is based on point estimates.^63,64^ Leave-one-out information criteria (LOOIC) and WAIC provide more accurate predictive performance estimates than AIC or DIC.^60^ Expected log predictive density (ELPD) measures predictive accuracy for comparing models in LOO.^65,66^ LOO calculates the ELPD from posterior samples, providing a Bayesian model comparison alternative to Bayes Factor and DIC.^67^ For model comparison, we used WAIC (ELPD-WAIC, P-WAIC and WAIC)^68–70^ and LOO-CV (ELPD-LOO, P-LOO and LOOIC).^62,70^ A lower value of WAIC and LOOIC indicates better model fit.^70^

#### Confounding assessment

Initially, non-parametric correlation matrices and the variance inflation factor were used for assessing potential multicollinearity between model predictors. Confounding was assessed using the 10% Change-in-Estimate Rule, classifying a variable as a confounder if its removal altered the effect estimate of other predictors by ≥10%.^71^

#### Interaction terms

Factors not selected during the initial screening were subsequently added one at a time to determine if their inclusion influenced the outcome, conditional on the existing model terms. Finally, we tested all possible two-way interactions among final predictors to evaluate the presence of effect modification, which were non- significant and excluded from final models.

#### Variable selection based on Least Absolute Shrinkage and Selection Operator

Least Absolute Shrinkage and Selection Operator (LASSO) is a regression method with an L1 penalty, useful for variable selection.^72^ LASSO simplifies models by selecting key variables, reducing overfitting and balancing bias and variance to improve prediction accuracy on new data.^73^ LASSO is useful for selecting important covariates, which is crucial when testing for threshold effects that could be obscured in models with many variables.^74^ Predictors for *E. coli* contamination levels in households’ drinking water were identified using LASSO regression, where variables with non-zero coefficients were considered important predictors. Most studies on LASSO focus on high-dimensional data.^75,76^ Conversely, LASSO can outperform stepwise regression even in low-dimensional settings,^77,78^ where the number of observations exceeds the number of predictors.^73^

We estimated rate ratios (RRs) with 95% credible intervals (CIs) from the final model. The Bayesian CI is a widely used measure of uncertainty in Bayesian inference.^79^ In the Bayesian analysis, 95% CI is statistically significant when CI does not include 1.^79^ Data analysis was conducted using R 4.0.4 (https://www.r-project.org/).

## Results

### Distribution of households

**Figure 2.**
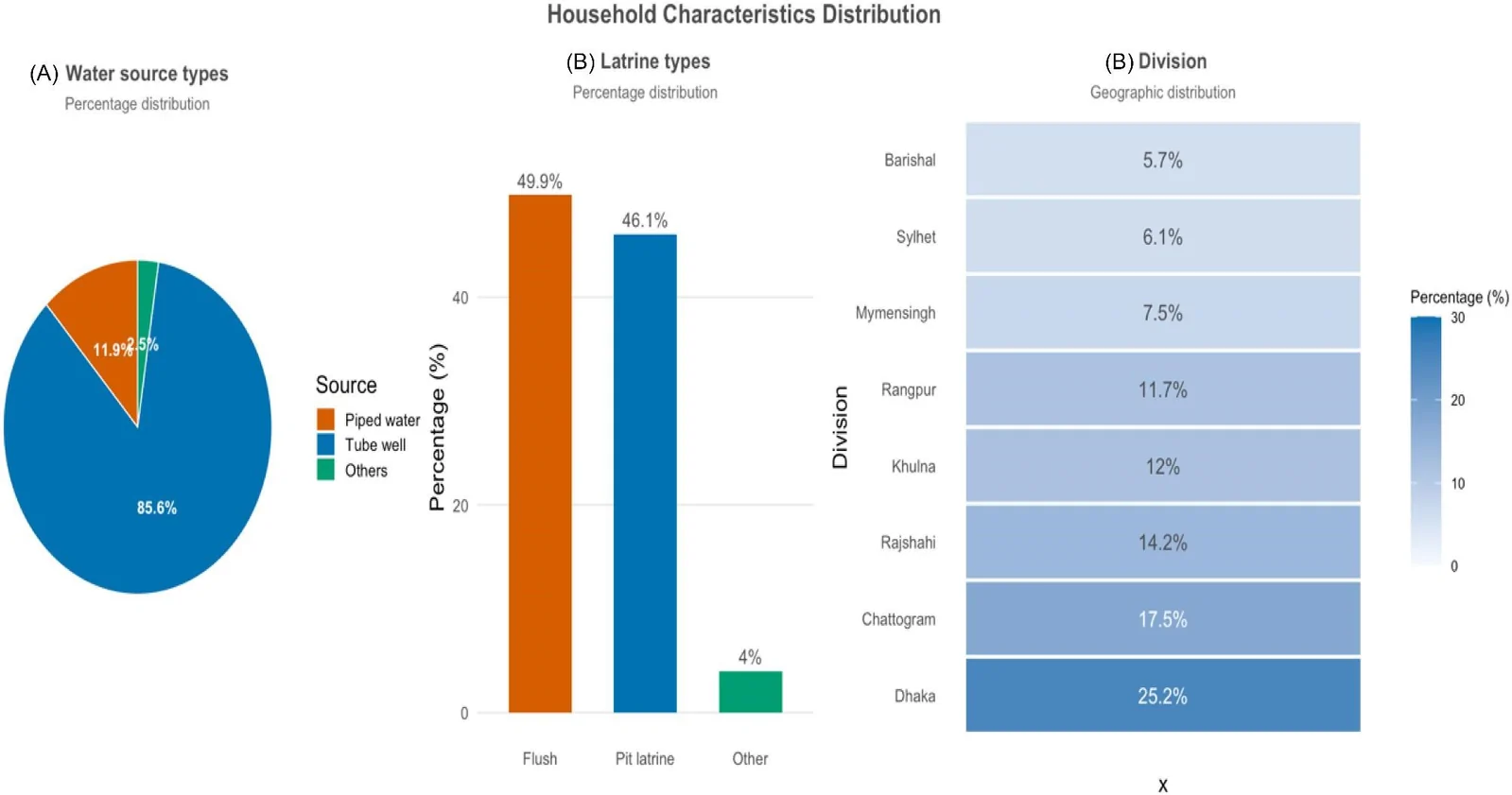
Percentage distribution of household characteristics: (A) water source types, (B) latrine types and (C) division.

Table 1 displays the percentage distribution of households according to different habitual and economic characteristics. Most households (25.2%) were from the Dhaka division, and the Barishal division had the lowest number of households (5.7%) (Figure 2C). Most households (77.8%) were from rural areas. More than one-half of the respondents had animals (56.7%). Slightly more than two-thirds (67.2%) of respondents reported that their water source was in their own yard. The majority of households (89.8%) did not treat their drinking water. According to wealth index, rich and poor households were almost equally represented (38.9% and 41.5%, respectively) and 19.7% were from the middle class. More than one-third (35.1%) of household heads had no record of education, while a similar amount (36.9%) had secondary level of education or higher. Among mothers, almost one-half (46.2%) had completed secondary education or higher, while only a small percentage (16.2%) had no formal education. Nearly one-half (46.4%) of households had 4–5 members, while a small group (19.5%) of households contained ≥6 individuals. Almost 56.2% of respondents were exposed to mass media.

**Table 1.** Characteristics of households enrolled in the MICS 2019

Covariate	Number (weighted)	(%)^a^
Division		
Barishal	348	5.7
Chattogram	1062	17.5
Dhaka	1532	25.2
Khulna	728	12.0
Mymensingh	455	7.5
Rajshahi	862	14.2
Rangpur	713	11.7
Sylhet	369	6.1
Residence		
Urban	1347	22.2
Rural	4722	77.8
Livestock ownership		
No	2620	43.2
Yes	3440	56.7
Location of water sources^a^		
Own dwelling	282	4.6
Own yard	4081	67.2
Elsewhere	1081	17.8
Treatment of drinking water		
No	5449	89.8
Yes	619	10.2
Gender of the household head		
Male	5268	86.8
Female	799	13.2
Wealth index		
Poor	2517	41.5
Middle	1194	19.7
Rich	2358	38.9
Household head education level		
No education	2133	35.1
Primary	1695	27.9
Secondary+	2241	36.9
Women education level^a^		
No education	985	16.2
Primary	1270	20.9
Secondary+	2804	46.2
Mass media exposure		
No	2660	43.8
Yes	3409	56.2
Household size		
<4	2066	34.0
4–5	2817	46.4
≥6	1186	19.5
Drinking water		
Piped water	723	11.9
Tube well	5194	85.6
Other	152	2.5
Pit latrine or septic tank ever been emptied		
Yes	2102	34.6
No	3967	65.4
Place of handwashing		
Dwelling	1038	17.1
Yard/plot	3650	60.1
Mobile object	612	10.1
No place	769	12.7
Toilet facility shared^a^		
Yes	1761	29.0
No	4207	69.3
Type of toilet facility		
Flush	3028	49.9
Pit latrine	2801	46.1
Other	240	4.0

MICS: Multiple Indicator Cluster Survey.

a Missing cases were kept as a category in calculations but are not included in the table.

The availability of pipeline drinking water was low (11.9%); the majority of households (85.6%) had tube wells for drinking water (Figure 2A). Nearly two-thirds (65.4%) of households had never emptied their septic tank or pit latrine. Around 12.7% of households did not have any place for washing hands, and most (60.1%) reported washing their hands in yards or plots; 17.1% of households had a place for handwashing within their dwelling. Slightly >85% of the dwellings were owned and the others were rented. Nearly one-third of households (29%) shared toilets. Furthermore, 49.9% and 46.1% of households had flush and pit latrine toilet facilities, respectively, while only 4% had other types of toilet facilities (Figure 2B).

### Feature selection

**Figure 3.**
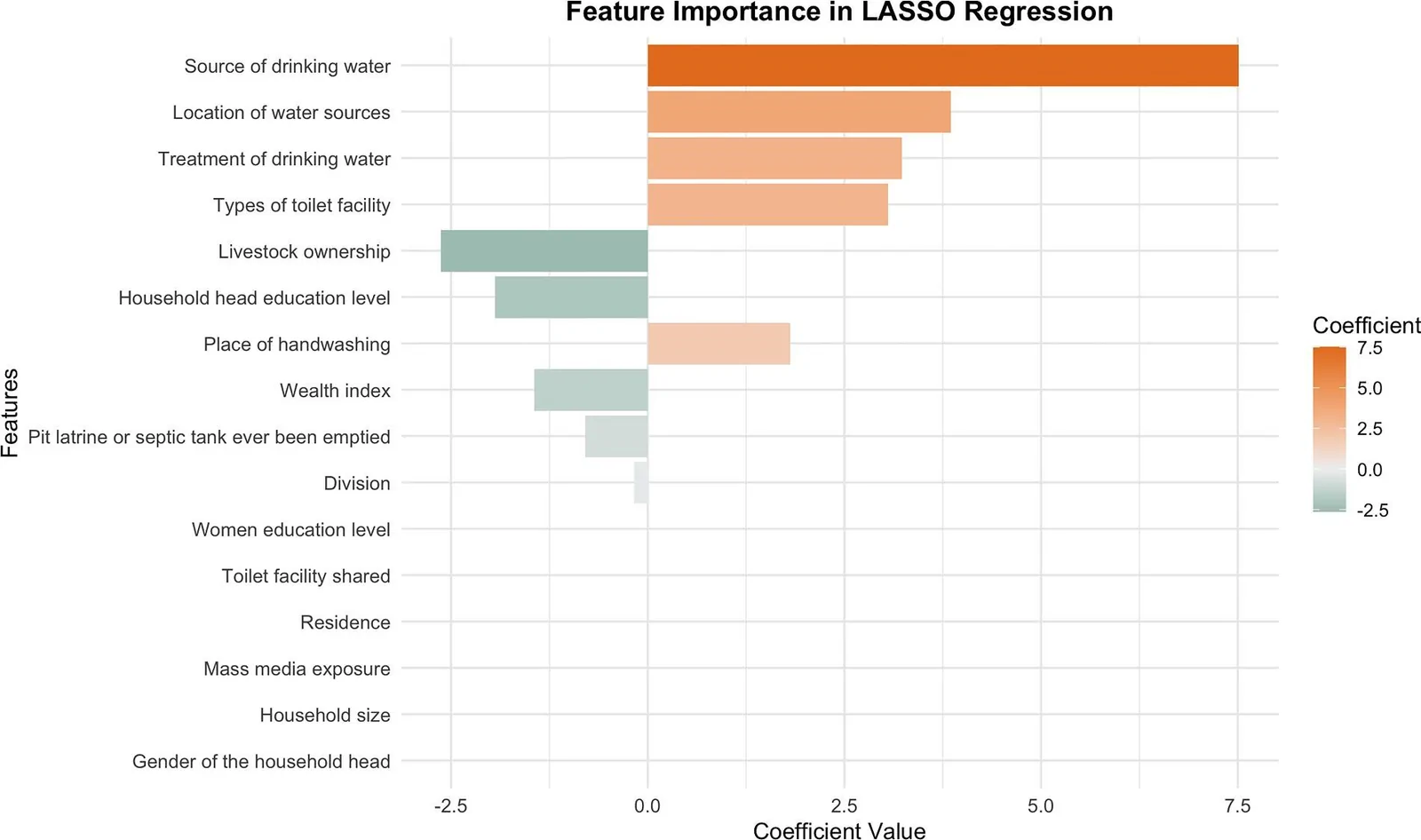
Variables selection by using the LASSO regression approach. The length and color of each bar represent the magnitude and direction (positive or negative) of the coefficient. Source of drinking water is the most important positive predictor, while livestock ownership is the most significant negative predictor count of *E. coli* contamination. LASSO: Least Absolute Shrinkage and Selection Operator.

In the context of LASSO regression, the key variables identified as significant predictors of *E. coli* contamination in household tap water in Bangladesh are presented in Figure 3. Variables include administrative division, livestock ownership, water source location, drinking water treatment practices, household head’s education level, mass media exposure, wealth index, type of drinking water source, history of pit latrine emptying, handwashing location and toilet facility type. Only variables with non-zero coefficient estimates were retained in the final model, ensuring the selection of the most relevant predictors.

### Model comparison

The BCGPR model exhibited lower WAIC (−472 425.9 vs 300 659) and LOO-CV (−472 429.2 vs 300 660.4) values compared with the BTPR model, indicating a superior model fit (Table 2). Our BCGPR model LOO-CV diagnostics indicate good stability, convergence and reliability because the Monte Carlo standard error (0.2) is small, ensuring precise ELPD estimation. The r_eff values (0.4–1.7) fall within an acceptable range, indicating a sufficient effective sample size. Additionally, all (100%) Pareto k values are <0.7, suggesting that no problematic data points are influencing the predictions that provide estimates, with relatively small variance and bias.^80^

**Table 2.** Bayesian Censored Regression Model estimates and 95% credible intervals for determinants of *E. coli* contamination in household drinking water in Bangladesh

	BTPR			BCGPR		
Variable		95% CI			95% CI	
Division	CRR	LL	UL	RR	LL	UL
Barishal (ref.)	1					
Chattogram	0.91	0.90	0.93	0.82*	0.79	0.84
Dhaka	1.09	1.07	1.10	1.01	0.97	1.03
Khulna	0.99	0.98	1.01	0.89*	0.86	0.91
Mymensingh	0.80	0.79	0.82	0.48*	0.46	0.51
Rajshahi	0.93	0.91	0.94	0.83*	0.81	0.86
Rangpur	0.56	0.55	0.57	0.80*	0.77	0.82
Sylhet	0.93	0.91	0.94	0.88*	0.85	0.92
Livestock ownership						
No (ref.)	1					
Yes	1.10	1.09	1.11	1.16*	1.14	1.17
Location of water sources						
Own dwelling (ref.)	1					
Own yard	1.11	1.07	1.13	1.14*	1.10	1.18
Elsewhere	1.17	1.15	1.20	1.17*	1.12	1.22
Treatment of drinking water						
No (ref.)	1					
Yes	0.93	0.92	0.95	0.95*	0.92	0.98
Household head education						
No education (ref.)	1					
Primary	0.94	0.93	0.94	0.99	0.97	1.01
Secondary and above	0.88	0.87	0.89	0.93*	0.91	0.94
Mass media exposure						
No (ref.)	1					
Yes	1.01	0.99	1.02	0.99	0.98	1.01
Wealth index						
Poor (ref.)	1					
Middle	0.99	0.98	1.01	0.93*	0.91	0.95
Rich	0.94	0.93	0.95	0.94*	0.92	0.96
Source of drinking water						
Piped water	1					
Tube well	0.89	0.88	0.91	0.90*	0.87	0.94
Other	1.10	1.07	1.13	1.33*	1.27	1.40
Pit latrine or septic tank ever been emptied						
No (ref.)						
Yes	1.03	1.02	1.04	1.07*	1.05	1.09
Place of handwashing						
Dwelling (ref.)						
Yard	1.13	1.11	1.14	1.22*	1.19	1.26
Mobile object	1.05	1.04	1.07	1.07*	1.04	1.11
No place	1.16	1.14	1.18	1.04*	1.01	1.07
Type of toilet facility						
Flush (ref.)						
Pit latrine	1.09	1.08	1.10	1.03*	1.01	1.05
Other	1.15	1.12	1.17	1.32*	1.28	1.37
Rank-normalized ${\boldsymbol{\hat{R}}} $ (each model parameters)	1.00			1.00		
Model fitness						
WAIC						
ELPD-WAIC	−150 329.52			236 213.0		
P-WAIC	997.63			773.8		
WAIC	300 659.04			−472 425.9		
LOO-CV						
ELPD-LOO	−150 330.24			236 214.6		
P-LOO	998.32			772.1		
LOOIC	300 660.48			−472 429.2		

BCGPR: Bayesian Censored Generalized Poisson regression; BTPR: Bayesian Tobit Poisson regression; CRR: count rate ratio; ELPD: expected log predictive density; LL: lower limit; LOOIC: leave-one-out information criteria; LOO-CV: leave-one-out cross-validation; RR: rate ratio; UL: upper limit; WAIC: Watanabe applicable information criterion. Significant at *p<0.05.

### Determinants of fecal contamination among household drinking water in Bangladesh

**Figure 4.**
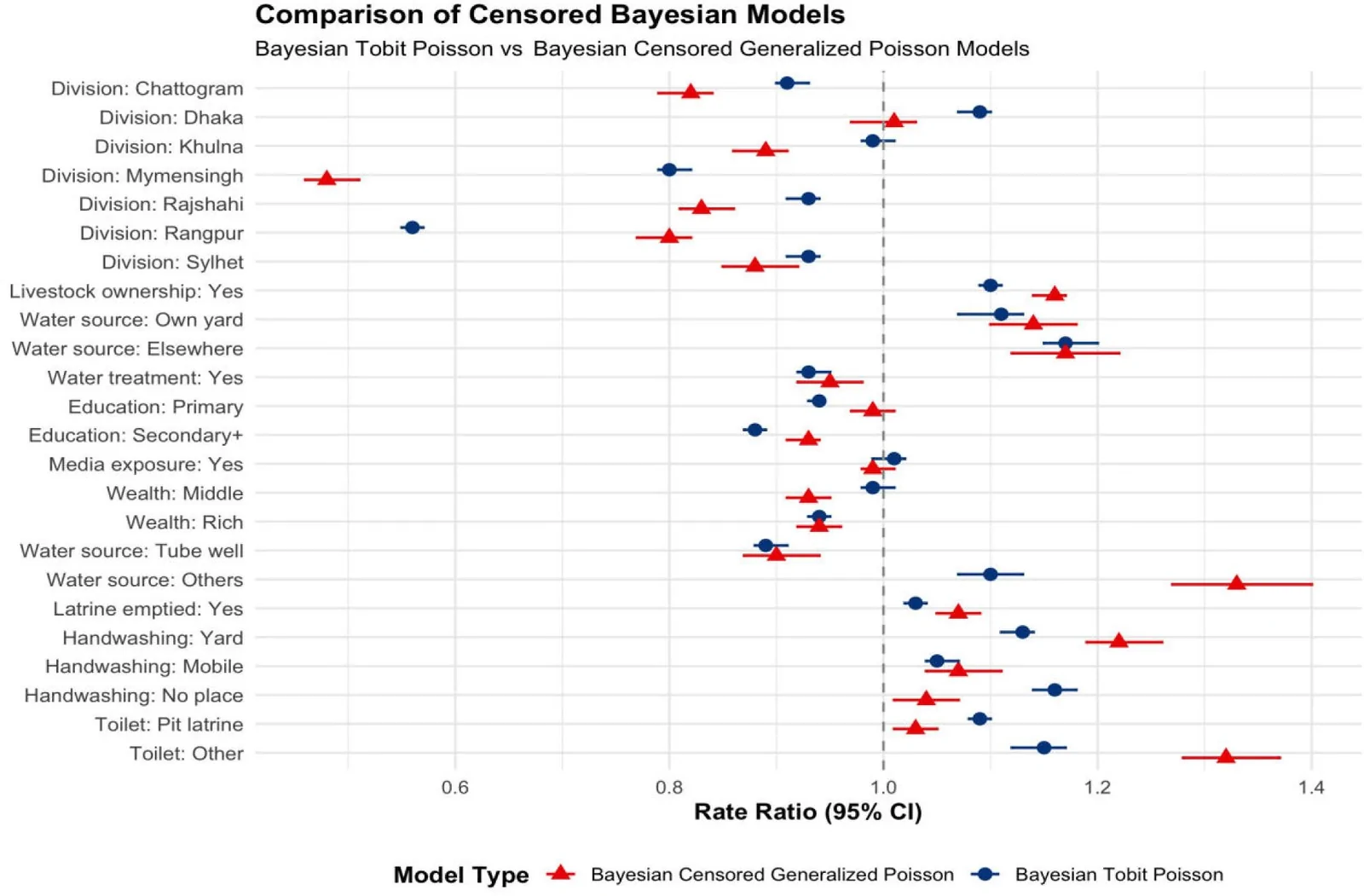
The forest plot presents the results of a comparison of censored Bayesian models for the determinants of *E. coli* contamination in drinking water.

According to the WAIC and LOOIC criteria, the BCGPR model provided a better fit for identifying factors associated with the count of *E. coli* in household drinking water. From Table 2, several variables were significantly associated with *E. coli* contamination in drinking water in Bangladesh. These include division, livestock ownership, location of water sources, treatment of drinking water, education level of the household head, wealth index, source of drinking water, whether the pit latrine or septic tank had ever been emptied, place of handwashing and type of toilet facility (Figure 4).

Chattogram, Khulna, Mymensingh, Rajshahi, Rangpur and Sylhet divisions had 18%, 11%, 52%, 17%, 20% and 12% lower expected counts of *E. coli* in household drinking water, respectively, compared with the Barishal division. Households that owned livestock had higher counts of *E. coli* in their drinking water compared with households that did not own livestock (Table 2).

The location of water sources was also significantly associated with *E. coli* contamination in tap water. Compared with households with water sources located within their own dwelling, those with water sources in their own yard and other locations were associated with 1.14 and 1.17 times higher *E. coli* contamination, respectively, in household tap water. Households that treated their drinking water had a 5% lower *E. coli* count compared with those that did not.

Additionally, households with heads having secondary education or higher had a lower rate of *E. coli* contamination in tap water (RR=0.93, 95% CI 0.91 to 0.94) compared with those with lower education levels. Households from upper socioeconomic classes had lower CFU levels of *E. coli* compared with those of poorer families.

The source of household drinking water is also statistically associated with the presence of *E. coli*. For instance, compared with piped water, households using tube wells had lower *E. coli* contamination levels in tap water (RR=0.90, 95% CI 0.87 to 0.94), while those relying on other water sources had 1.33 times higher *E. coli* contamination (RR=1.33, 95% CI 1.27 to 1.40). Households with handwashing facilities located in the yard, on a mobile object, or having no designated place, had 1.22, 1.07 and 1.04 times higher *E. coli* contamination in tap water, respectively, compared with those with handwashing facilities inside the dwelling. Households using pit latrines and other types of toilet facilities had 1.03 and 1.32 times higher *E. coli* contamination in tap water, respectively, compared with those with flush toilet facilities.

#### MCMC convergence and mixing diagnostics for BCGPR

**Figure 5.**
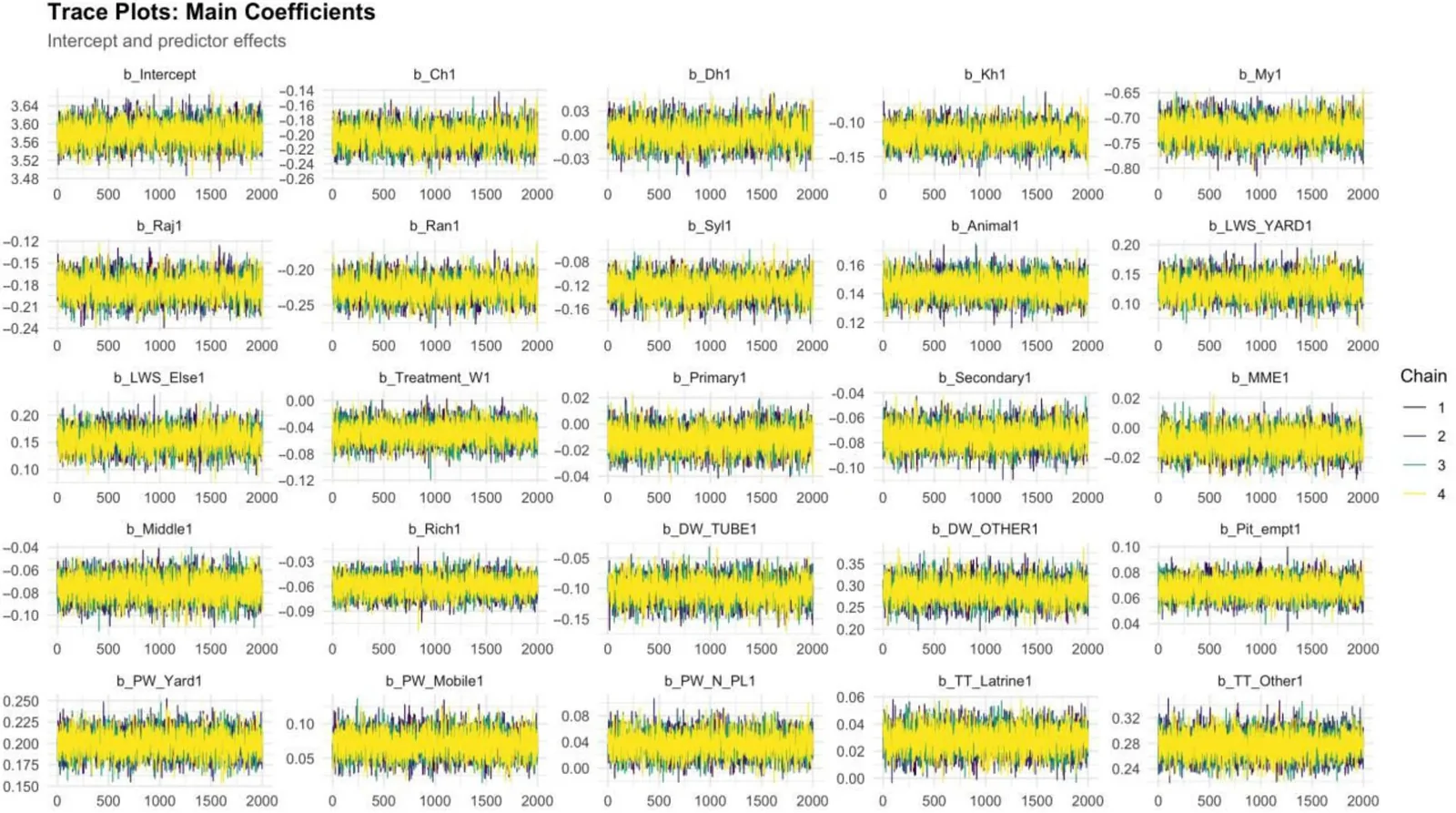
Trace plots for MCMC convergence diagnostics of a BCGPR model. The figure shows four different MCMC chains (colored differently: light grey, purple, blue green and bright yellow). BCGPR: Bayesian Censored Generalized Poisson regression; MCMC: Markov Chain Monte Carlo.

The trace plots indicate that the BCGPR model has achieved good MCMC convergence and mixing, meaning the posterior estimates for the parameters are stable and valid (Figure 5). The trace plots suggest that the estimates for the model parameters are stable across iterations. For further convergence assessment, we used rank-normalized ${\boldsymbol{\hat{R}\ }}$and estimated its value as ${\boldsymbol{\hat{R}}}$=1 (for each model parameters), which indicates that the MCMC chains have fully converged. This indicates that the Markov chains are well mixed, and their stationary distributions are nearly identical across different chains. Additionally, autocorrelation plots show rapid decay toward zero within the first few lags, indicating good chain mixing, minimal autocorrelation and adequate convergence for the reliable BCGPR model (Supplemental Figure 2).

**Figure 6.**
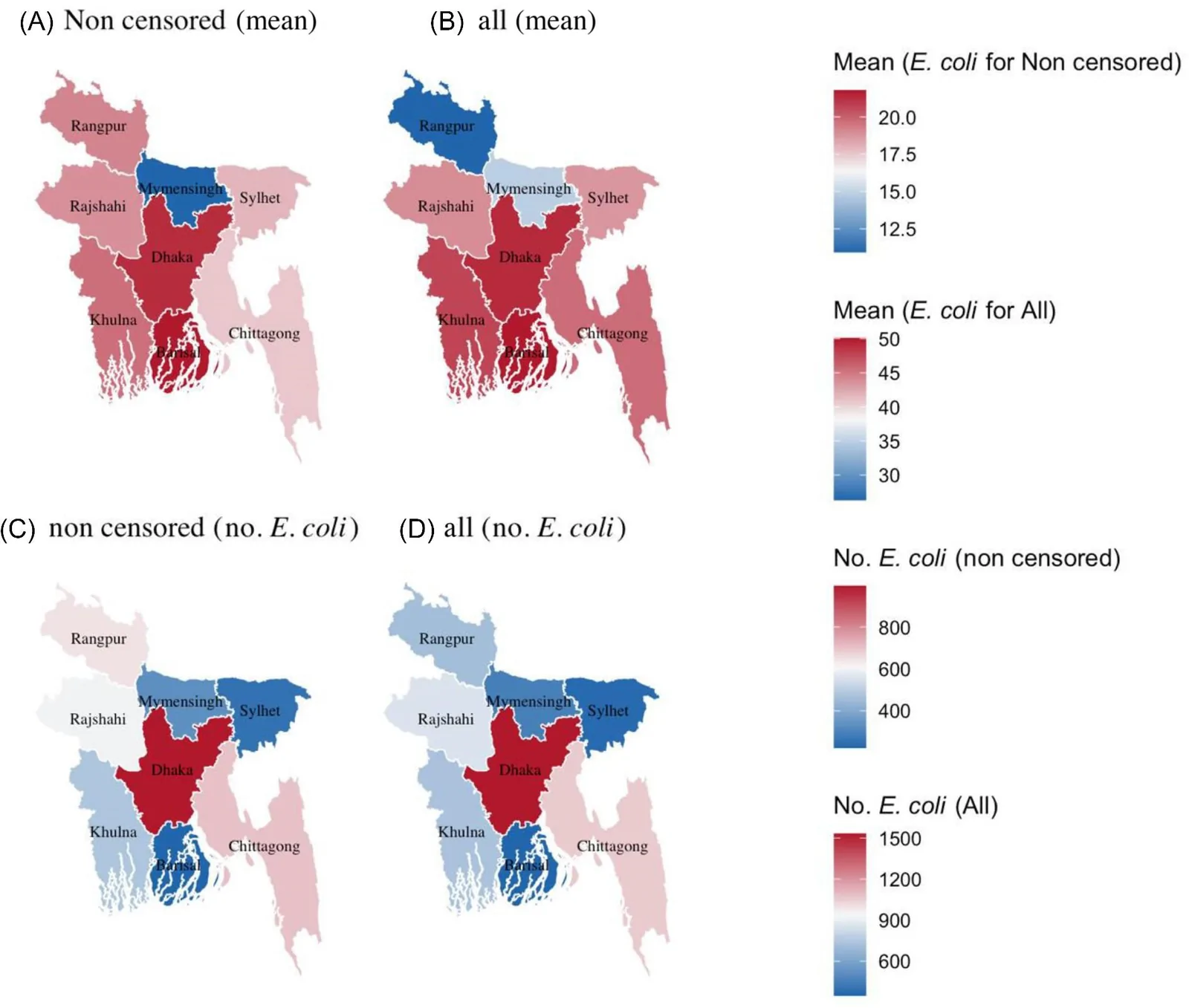
Geographic distribution of *E. coli* contamination in Bangladesh by division: (A) non-censored (mean); (B) all samples (mean); (C) non-censored (no. *E. coli*); and (D) all samples (no. *E. coli*).

For complete cases, the Barishal and Dhaka divisions showed the highest average contamination levels, while Mymensingh had the lowest (Figure 6A). For all cases, including censored observations, Barishal had the highest *E. coli* contamination levels, whereas Rangpur had the lowest values among all divisions (Figure 6B).

## Discussion

Using data from the Bangladesh MICS 2019, we investigated potential risk factors associated with levels of *E. coli* in households’ drinking water using Bayesian models for censored count data. Our overarching goal was to identify regions and socioeconomic groups that should be prioritized to improve water and sanitation practices. Our findings demonstrate that division, livestock ownership, the location of water sources, the treatment of drinking water, household head education, wealth index, the source of drinking water, the place used for handwashing and the type of toilet facility were the most significant factors associated with the levels of *E. coli* in drinking water.

Type and location of water source were important predictors of *E. coli* contamination levels. Households that sourced water from a yard and other sources had higher levels of *E. coli* in tap water than others that sourced from their own dwelling. This is consistent with earlier findings in Peru^81^ and Thailan^82^ and shows that water from reservoirs, like buckets, is more likely to contain *E. coli*. Additionally, tube well water had significantly lower *E. coli* counts than drinking water from piped sources. These results may be explained by intermittent piped water flows, which can facilitate contamination during supply interruptions. Continuous (24/7) tap water supply has been shown to have fewer contamination events and lower indicator bacteria levels compared with intermittent piped water systems.^83^ Intermittent piped water increases disease risk due to contamination in unpressurized pipes, recontamination during storage, use of unsafe alternatives and limited water availability for hygiene.^84^ Moreover, fecal contamination can affect all water sources, including piped water.^85,86^ Consequently, piped systems in developing countries may not guarantee safe drinking water.^83,87^ In Bangladesh, only about 12% of areas have piped water (32% urban, 1% rural), and these systems often face disinfection failure, leakage, interruptions and low pressure.^88^ A previous study in Bangladesh found that piped systems exhibited both disinfection and distribution failures.^89^ By contrast, most tube well samples were uncontaminated, indicating safe aquifers, with contamination primarily occurring at the secondary (household) level.^90^ Piped water had higher contamination in rural areas, with no difference for others.^91^ Unimproved water sources include unprotected wells, springs and surface water,^92^ each of which produces higher contamination levels.^93^ The virulence genes of *E. coli* in Tanzanian household water have been reduced through improved water sources.^94^

Additionally, toilet facility type was significantly associated with *E. coli* contamination levels in households. Pit latrines and other toilet types displayed higher *E. coli* levels in tap water compared with flush toilets. The minimal effect size observed here indicates that, after adjusting for wealth, education, water source and other covariates, the contribution of pit latrine type to the outcome is very small. Statistically significant yet small effects for risk factors are increasingly common in public health research.^95^ Interestingly, models for categorical data did not identify a similar association after adjusting for confounding.^23^ This emphasizes the advantage of modeling the actual counts of bacteria as opposed to categorized outcomes. Importantly, the link between toilet facilities and *E. coli* contamination levels in drinking water has been demonstrated.^96^

Compared with handwashing in dwellings, all other handwashing locations (yard, mobile or no designated place) had increased *E. coli* contamination levels in household water. Running tap water in the household significantly reduces *E. coli* contamination in drinking water, as well as in water for handwashing.^97^ Additionally, handwashing with soap reduces *E. coli* transmission risks from fomites during food preparation.^98^

There was a negative association between wealth index, household head education and *E. coli* levels in tap water in Bangladesh. Households with a higher wealth index (e.g. rich households) or higher education levels (e.g. secondary and higher education) had lower levels of *E. coli* in drinking water. Previous studies have shown that a higher asset index score in household possessions is linked to improved water sources and reduced *E. coli* contamination in drinking water.^99,100^ Socioeconomic status and lack of formal education have been linked with a higher level of fecal contamination.^101,102^ This is consistent with an earlier investigation conducted in Peru, which observed that household members with higher education were less likely to have *E. coli* in their drinking water.^81^ Higher education levels are associated with better water management and hygiene practices, which reduce the likelihood of *E. coli* contamination in drinking water sources.^103^

Livestock ownership was significantly associated with *E. coli* levels, where households with livestock had higher bacteria concentrations compared with households without livestock. This aligns with a previous study conducted in Bangladesh, which analyzed the same dataset using risk categories as outcome.^23^

There was a significant association between division and *E. coli* levels in drinking water. Households in the Mymensingh division had the lowest levels of *E. coli* in tap water compared with those in Barishal, followed by Rangpur, Chattogram, Rajshahi, Sylhet and Khulna. This regional difference can also be explained from a geographical perspective. For example, Barishal, a low-lying coastal division only 1.22 m above sea level with many river tributaries, is highly prone to flooding, cyclones and storm surges.^104–106^ Remote areas of Bangladesh, particularly the disaster-prone Barisal division, lag in sanitation access, with less than one-half of the population using improved facilities.^107^ On the contrary, Mymensingh has been reforming its water management strategies to address pollution and climate-related vulnerabilities.^108^ Strengthening resilient water, sanitation and hygiene infrastructure is crucial to protect health amid flooding and cyclones and to understand its links with drowning risk in this vulnerable region.^107^ In a previous study in Bangladesh, *E. coli* was detected in 18.3% and 11.8% of milk samples from markets and dairy farms, respectively, in Chittagong.^109^ Significant regional disparities in the use of *E. coli*-contaminated water among household members was demonstrated. Households in Barisal and Khulna were 3.41 and 1.06 times more likely, respectively, to use highly contaminated drinking water compared with those in Rangpur.^23^ This suggests that there are discrepancies in the levels of fecal contamination in Bangladesh, as observed in our study. Potential reasons include variations in sanitation infrastructure, differences in water source quality, inconsistent implementation of hygiene practices, population density, public health initiatives and access to clean water. In the MICS 2019, Barisal, with 94.0% tube well-reliant population and 55.1% off-premises water sources, has the highest proportion of *E. coli*-free water access (84.1%) at the Point of Collection, but the lowest (9.7%) at the Point of Use.^43^

Our findings demonstrate that rural location was not significantly associated with the number of *E. coli* (CFU per 100 mL) in household drinking water. In many low-income urban settings, limited space and resources often require multiple households to share sanitation facilities, circumstances that may increase the risk of diarrhea and complicate arrangements for cleaning and maintenance.^110–112^ High levels of fecal contamination have been detected across multiple residential compartments in Dhaka, indicating that all areas of the city are vulnerable to contamination regardless of socioeconomic status.^15^ Living in rural households was linked to a 10% higher risk of water contamination than urban ones, after adjusting for source, sanitation, wealth and other factors.^113^ Further, in agreement with our findings, coliform levels in water reservoirs near urban and industrial areas were significantly higher than those in remote areas.^114^ In a study conducted in Ethiopia, children from rural areas were four times more likely to be infected by *E. coli* O157 than their urban counterparts.^115^ Importantly, in our study, many factors independently associated with *E. coli* levels in drinking water were not deemed relevant in the multivariable analysis. This underscores the benefits of conducting a detailed epidemiological investigation to identify the most critical elements linked to the outcome of interest.

These findings highlight critical areas for intervention to improve water quality and public health in Bangladesh, directly supporting Sustainable Development Goals (SDGs), particularly SDG 6 (Clean Water and Sanitation) and SDG 3 (Good Health and Well-being). Policies should focus on enhancing educational opportunities, particularly for household heads, as higher education levels correlate with lower *E. coli* contamination. Improved infrastructure such as tube wells could significantly reduce fecal contamination of tap water. Interventions should account for household practices such as proper handwashing and consistent toilet maintenance. In Barishal, where higher contamination levels were observed, targeted hygiene campaigns led by community health workers should be stimulated. Educational materials using visuals, pictograms and culturally tailored storytelling can improve engagement and effectiveness in low-literacy communities.

### Strengths and limitations

The current study has both strengths and limitations. Its primary strength lies in the use of nationally representative data, encompassing a substantial sample size of 6069 households from the MICS 2019. Another strength lies in the utilization of regression models for count data and identification of the best one. Despite the aforementioned strengths, the findings of our study should be considered in the context of its limitations. Our dataset missed important variables known to contribute to fecal contamination of water sources, such as soil type, weather, agricultural and animal production activities in the proximities, as well as waste disposal protocols. Additionally, because this study relied on secondary data and employed a cross-sectional design, it was not possible to establish cause-and-effect relationships. Previous research using the Bangladesh MICS 2019 highlighted gaps in capturing the full complexity of urban slum environments and their impact on maternal and child nutrition across Asia and the Pacific.^116^ This suggests that while the MICS provides valuable nationally representative data, it may not fully represent conditions in urban slums, limiting the generalizability of findings to these vulnerable populations.

### Conclusion

We determined socioeconomic characteristics contributing to the levels of *E. coli* in household drinking water in Bangladesh in 2019 using Bayesian models for count data. In summary, households located in the Barishal division, led by individuals of low educational background, and wherein drinking water was not sourced from tube wells or pipes, had increased *E. coli* counts in drinking water compared with their counterparts. Households with pit latrines or other types of toilet facilities, handwashing places other than the dwelling, livestock ownership and water sources located in the yard or elsewhere, were more likely to have *E. coli* contamination than their counterparts. These findings highlight the need for targeted interventions, including prioritizing the expansion of flush toilet facilities, increasing community awareness on hygiene practices and subsidizing safe water storage containers. Furthermore, we recommend longitudinal and intervention-based research to better understand causal relationships and the effectiveness of such measures in reducing household water contamination.

## Supplementary Material

ihaf138_Supplemental_File

## Data Availability

In this study, we used data from the Bangladesh MICS 2019, which is available from https://mics.unicef.org/surveys?display=card&f[0]=round:53&f[1]=region:2551&f[2]=region:2521.
