# Supplementary material for: Bayesian modeling of Escherichia coli contamination in household drinking water in Bangladesh: evidence from the Multiple Indicator Cluster Survey 2019
Source: Int Health. 2025 Dec 5;18(5):742–57. doi: 10.1093/inthealth/ihaf138 (PMC13530297; doi:10.1093/inthealth/ihaf138)
Supplement: Supplementary file 1 [file ihaf138_supplemental_file.docx]

| **Variable** | **Type** | **Categories / Coding** | **Source** |
| --- | --- | --- | --- |
| Division | Categorical | Barishal, Chattogram, Dhaka, Khulna, Mymensingh, Rajshahi, Rangpur, Sylhet | MICS 2019 |
| Residence | Categorical | Urban, Rural | MICS 2019 |
| Livestock ownership | Binary | Yes, No | MICS 2019 |
| Location of water source | Categorical | Dwelling, Yard, Elsewhere | MICS 2019 |
| Source of drinking water | Categorical | Piped, Tube well, Other^1^ | MICS 2019 |
| Treatment of drinking water | Binary | Yes, No | MICS 2019 |
| Types of toilet facility | Categorical | Flush, Pit latrine, Other | MICS 2019 |
| Toilet facility shared | Binary | Yes, No | MICS 2019 |
| Pit latrine or septic tank ever been emptied | Binary | Yes, No | MICS 2019 |
| Place of handwashing | Categorical | Dwelling, Yard/plot, Mobile, None | MICS 2019 |
| Household size | Categorical | <4, 4–5, 6+ | MICS 2019 |
| Wealth index | Categorical | Poor, Middle, Rich | MICS 2019 |
| Household head education level | Categorical | No education, Primary, Secondary+ | MICS 2019 |
| Woman’s education | Categorical | No education, Primary, Secondary+ | MICS 2019 |
| Mass media exposure | Binary | Yes, No | MICS 2019 |

**Supplemental**

Table 1. Summary of independent variables

^1^The “Other” category included less common drinking water sources such as protected or unprotected dug wells and springs, rainwater, cart or tanker water, water kiosks, surface water, bottled water, and any other reported source

**Figure 1. Bayesian Overdispersion Test Indicating Violation of the Poisson Assumption**

**Figure 2. Autocorrelation Diagnostics for the BCGPR Model**
